# Radiation-induced neuropathies in head and neck cancer: prevention and treatment modalities

**DOI:** 10.3332/ecancer.2020.1133

**Published:** 2020-11-03

**Authors:** Patrick Azzam, Manal Mroueh, Marina Francis, Alaa Abou Daher, Youssef H Zeidan

**Affiliations:** 1 Department of Anatomy, Cell Biology and Physiology, Faculty of Medicine, American University of Beirut, Beirut 1107 2020, Lebanon; 2 Department of Radiation Oncology, American University of Beirut Medical Center, Beirut 1107 2020, Lebanon

**Keywords:** radiation, neuropathy, head and neck cancer, treatment, prevention

## Abstract

Head and neck cancer (HNC) is the sixth most common human malignancy with a global incidence of 650,000 cases per year. Radiotherapy (RT) is commonly used as an effective therapy to treat tumours as a definitive or adjuvant treatment. Despite the substantial advances in RT contouring and dosage delivery, patients suffer from various radiation-induced complications, among which are toxicities to the nervous tissues in the head and neck area. Radiation-mediated neuropathies manifest as a result of increased oxidative stress-mediated apoptosis, neuroinflammation and altered cellular function in the nervous tissues. Eventually, molecular damage results in the formation of fibrotic tissues leading to susceptible loss of function of numerous neuronal substructures. Neuropathic sequelae following irradiation in the head and neck area include sensorineural hearing loss, alterations in taste and smell functions along with brachial plexopathy, and cranial nerves palsies. Numerous management options are available to relieve radiation-associated neurotoxicities notwithstanding treatment alternatives that remain restricted with limited benefits. In the scope of this review, we discuss the use of variable management and therapeutic modalities to palliate common radiation-induced neuropathies in head and neck cancers.

## Background

Head and neck cancer (HNC) is the sixth most common human malignancy accounting for 3% of all cancers, with 650,000 cases and 330,000 deaths per year [1, 2]. HNC is a disease involving heterogeneous regions in the head and neck area such as the pharynx, larynx, paranasal sinus, nasal cavity, oral cavity and salivary glands. There are histopathological and molecular differences between these cancers, along with their treatment modalities and outcomes. About 90% of cases occurring in these anatomically complex regions are squamous cell carcinomas, which are diagnosed at a late stage when clinical symptoms arise. Tumorigenesis in HNC has genetic and environmental roots. Long-term tobacco and alcohol consumption along with high-risk types of human papillomavirus were found to be the main oncogenic risk factors.

The treatment approaches of HNC involve surgery, radiotherapy (RT), chemotherapy, immunotherapy or combinational modalities. Around 80% of HNC patients undergo RT at least once during their course of the illness as primary or adjuvant treatment [3]. RT is known to inhibit the proliferation of tumour cells through different molecular mechanisms. Despite its effectiveness, radiation may induce collateral damage to neighbouring uninvolved normal tissues, exhibiting acute (weeks to months) or late (months to years) toxicities. The latter could severely impair patients’ quality of life (QoL) and even worsen the prognosis. While RT has greatly evolved in the past couple of decades, no technology can completely protect healthy tissues from radiation damage. HNC patients still have to cope with treatment-related symptoms, despite increased survival owing to novel advanced treatment protocols and research.

Patients with HNC undergoing radiotherapy are at great risk of developing neuropathies due to the abundance of the nervous tissues in the head and neck region. Radiotherapy can have deleterious and detrimental damaging effects on the nervous system because of the limited capacity for repair and regeneration of the nervous tissues. While not as common as other adverse effects in HNC, neuropathies leave a long-lasting compromise in the patients’ well-being. Radiation-induced neuronal damage in HNC manifests mainly as sensorineural hearing loss (SNHL) and alterations in taste and smell sensory functions (dysgeusia and dysosmia, respectively). Injuries to the brachial plexus and cranial nerves were also reported, along with optical neuropathies and trouble in swallowing (dysphagia) and speaking (dysphonia). Understanding neuropathies emanating from HNC radiotherapy is essential for improving patients’ quality of life, due to the irreversible nature of such injuries.

In this review, we discuss molecular mechanisms underlying radiation mediated neuropathies, followed by pathological aspects in head and neck cancer along with the available radioprotection and treatment methods.

### Molecular pathophysiology of radiation-induced neuronal injury

Radiation-induced neuronal injury is a multifactorial pathogenesis with acute and long-term onsets. Acute symptoms like drowsiness, nausea and headaches are usually reversible and may resolve spontaneously. However, long-term side effects such as radionecrosis, white matter abnormalities and cognitive impairments are progressive and often permanent. Radiation-induced late brain injuries are irreversible changes that occur 6 months after radiotherapy, characterised by morphological damage as demyelination, gliosis and abnormal vascularisation [4]. Similarly, peripheral nerve radiation insult is defined by altered axonal growth along with perineurium formation [5, 6]. On the cellular level, RT targets numerous molecular entities such as the integrity of the DNA molecule, oxidative stress, inflammatory cascades and synaptic activity. Ultimately, molecular damage leads to apoptosis, neuroinflammation and altered neuronal function, the main hallmarks of radiation-induced neuropathies in HNC.

### IR and neuronal apoptosis

Neurons are differentiated postmitotic cells with reduced ability to repair radiation-mediated DNA damage. Consequently, a slow accumulation of radiation-induced DNA adducts in the neuronal genome leads to apoptosis [7]. Neuronal tissues are very heterogeneous due to differences in localisation, types of cells (neurons and neuroglia) and cell–cell interaction mechanisms. Thus, there is no homogenous response to ionising radiation, and neural substructures have large variabilities in molecular reaction.

Many studies have established that neuronal apoptosis is dose-dependent and occurs shortly after RT [8]. A dose of 2 Gy was able to induce apoptosis in postmitotic cortical neurons as early as 4 hours post-irradiation. Therefore, neurons were shown to be radiosensitive in contrast to astrocytes which remain radioresistant at 32 Gy [9]. Total dose administration and delivery methods were found to have an interesting impact on neurons’ fate. For instance, fractionating the doses to allow DNA repair in between fractions proved to be a good strategy to reduce cell death in neurons [9]. Interestingly, a low dose of radiation (0.2 Gy) did not affect whatsoever reactive oxygen species (ROS) levels or neuronal synapses. Such low doses result in mitochondrial fusion and the expression of complexes I and III, promoting an adaptive cellular mechanism rather than cell death [10].

The key behind neuronal death is indeed direct or indirect radiation-induced ROS upregulation. Modulating oxidative stress levels by administration of free radicals scavengers as melatonin, rhubarb and kukoamine rescued neurons from radiation-mediated apoptosis [11–13]. In addition, tetracyclines derivates and ceftriaxone relieved neurons from oxidative stress and subsequent apoptosis [14]. Irradiated cell-conditioned medium culture promoted oxidative stress and cell death, which were alleviated if neurons were co-cultured with glial cells [15–17]. Furthermore, metallothionein protected neurons and astrocytes from ionising radiation potentially through an antioxidant mechanism [18]. Thus, promoting free radical scavenging rescued neurons from cell death post-irradiation.

Radiation-induced apoptosis was found to be p53-, Bax- and ATM-dependent in most neuronal structures, while the role of caspase-3 remains unclear [19–23]. Similarly, cell death in glial structures was found to be p53-dependent [24]. In sensory ganglion rat cells, the ATM/p53 pathway did not induce apoptosis but mediated neuronal entry into the G0-G1 transition phase by increasing p21 and cyclin-D [25]. Calcium ions were also shown to modulate RT-induced cell death in immature dorsal ganglion root [26, 27]. Whether calcium ions rescue neurons by abrogating p53-dependent apoptosis remains to be elucidated. On another note, multiple studies suggest the implication of c-Jun in radiation-induced apoptosis of different neuronal substructures [28, 29].

Targeting apoptosis-related pathways could be a therapeutic modality in rescuing neurons from RT-induced death. For instance, minocycline via its targeting to AMPKα1 inhibited neuronal apoptosis by promoting autophagy [14, 30]. Similarly, administration of GSK3ß and MDM2 inhibitors suppressed p53 accumulation and halted radiation-mediated cell death in hippocampal HT-22 cells [31]. Furthermore, NFAT3/c4 inhibition halted excitotoxicity induced apoptosis after radiation exposure in hippocampal neurons [32].

### IR and neuroinflammation

The main hallmark for neuroinflammation is the occurrence of radiation-induced fibrosis (RIF) which is a late, unavoidable complication of radiation injury. RIF occurs between 4 and 12 months after therapy, and its pathogenesis is well established. Following injury and apoptosis, tissues secrete chemoattractants known as proinflammatory cytokines, such as TNFα, IL-1ß, COX2 and AP-1 [8, 33]. These molecules activate nonspecific inflammatory cascades and promote neutrophils and monocytes recruitment to the site of injury. In such circumstances, neutrophils are engaged via increased ICAM-1 (Intercellular Adhesion Molecule 1) and PECAM-1 (Platelet endothelial cell adhesion molecule-1) expression on endothelial cells [4, 34]. In fact, nerve injury to the lumbar root in rats increases ICAM-1 and PECAM-1 immunoreactivity along with the invasion of lymphocytes into the spinal parenchyma [35]. Afterwards, monocytes are transformed into PDGF (Platelet-derived growth factor) secreting M2-macrophages, promoting the recruitment of stromal fibroblasts along with the differentiation of circulating mesenchymal cells. Through TGF-β (Transforming growth factor beta) secreted by M2-macrophages, stromal fibroblasts are differentiated into myofibroblasts that produce a surplus of extracellular matrix components such as collagen, along with inhibition of extracellular matrix-degrading enzymes. Thus, fibrotic areas are generated, leading to susceptible loss of function or even tissue atrophy [36].

A similar mechanism is initiated in the central nervous system where along with lymphocytes recruitment, there is an activation of microglia. This process acts through the NF-κb pathway coupled with the upregulation of microglial MHC and CD68. Injured neurons contribute to the inflammatory process by secreting HMGB1 (High mobility group box 1) and calreticulin which are ligands to microglia, thus promoting phagocytosis of both damaged and healthy neurons. This phenomenon is accompanied by the recruitment of peripheral macrophages through CCL2-CCR2 chemoattraction [4]. Eventually, persistent microglial activation will lead to amplified neuronal damage in the long term [8, 37]. Managing chronic neuroinflammation could alleviate a substantial part of the neuropathies observed following RT.

Peripheral nerves display electrophysiological changes in early phases of RIF pathogenesis. The pathological process includes axonal injury and demyelination, followed by a pronounced fibrotic tissue around nerve trunks and decreased vascular supply. Many risk factors can lead to such damage including a large total dose (>50 Gy to plexus, >60 Gy to cranial nerves), a large dose per fraction > 2.5 Gy, a considerable radiation field where nerve fibres are included and patients’ comorbidities [34].

### IR and alterations in neuronal function

While neurons can withstand small doses of radiation [10], acute and long-term morphological and electrophysiological changes occur in neuronal tissues with large doses. In fact, intrinsic neuronal membrane alterations were noted 3 months after exposure, hinting a long-term effect of radiation [38]. Dendritic architecture is compromised following a 10 Gy irradiation in hippocampal neurons, marked by a decrease in branching, length and density of dendritic spines, along with changes in synaptic protein levels [39]. Changes in synaptic transmission were recorded in the CA1 region of the hippocampus after radiation, characterised by the downregulation of spike generation, excitability and synaptic efficacy. Furthermore, radiation exposure resulted in an acute decline in excitatory NMDAR (N-methyl-D-aspartate receptor) receptors with a concurrent increase of inhibitory GABA (γ-aminobutyric acid) receptors corresponding with suppressed long-term potentiation (LTP) [40]. In addition, radiation suppressed the expression of Arc, an essential protein in sustaining LTP and spatial memory consolidation [41]. Conversely, some studies mentioned a dramatic increase in synaptic activity after irradiation leading to neuronal excitotoxicity [32]. Intriguingly, low doses of radiation (0.1–0.5 Gy) increased synaptic excitability along with dendritic spines and neural outgrowth coupled to a reduced mitochondrial function [42, 43]. There is also growing evidence that low radiation dose confers to neurons a radioadaptive mechanism manifested in an upregulation of antioxidant enzymes and anti-inflammatory cytokines [44].

Overall, unattended imbalance in ROS levels due to radiation exposure may be behind the observed ‘domino effect’ in these neuropathies. From apoptosis to radiation-induced fibrosis, and impaired neuronal function, this pathological cascade needs to be halted in its earliest stages for patients to have better survival chances.

### Sensory dysfunctions and radioprotection

Head and neck cancer radiation-induced neuropathies can affect fundamental sensory functions. This can manifest as hearing loss/decline, taste and smell alterations, along with different neuropathies involving the brachial plexus and cranial nerves. Thus, it is of utmost importance to address these issues in preclinical experiments in order to tackle the pathological mechanisms and develop curative strategies.

### Sensorineural hearing loss

Sensorineural hearing loss is a common neurological complication that emerges following radiotherapy in HNC patients. A higher risk of developing SNHL is noted when the inner cochlea is within the radiation field of primary tumour sites [45]. Auditory organs that are most affected by radiation injury are the stria vascularis and organ of Corti of the cochlea. Following irradiation, recruitment of macrophages and inflammatory cells is ensured by the stria vascularis, thus triggering inflammation and ROS imbalance in the auditory milieu. This promotes chronic injury to the auditory pathway manifested in p53-dependent apoptosis of the outer and inner auditory hair cells [46]. Therefore, radiation-induced auditory insult manifests as hearing loss/decline, potential ossification of the inner ear fluid space and even inflammation-mediated oedema in the cochlear nerve [47]. Para-cochlear structures as the spiral ganglion neurons may also be injured, resulting in abnormal auditory brainstem responses (ABR) [46].

High-frequency hearing (above 4 kHz) was mainly affected in HNC patients receiving radiotherapy, whereas speech frequency (below 4 kHz) was less damaged. Incidence of SHNL increased progressively with time and was found to be dose-dependent and permanent [46]. A randomised prospective trial, including 110 patients noted no significant differences in cochlear sparing between conventional radiotherapy and intensity-modulated radiotherapy (IMRT). IMRT is an advanced technology that conforms radiation dose to the shape of the tumour, thereby sparing normal tissues from radiation [48]. Both groups had hearing loss superior to 10 db, while no discrepancies were found in balance assessment, ototoxicity, QoL and progressive hearing impairment. These results could be explained by the considerable number of patients who withdrew from the study leading to a reduction in its statistical power [49]. Another suggestion could be the radiosensitivity of the auditory apparatus regardless of the treatment technique received by patients.

Many experimental studies have been conducted *in vitro* and *in vivo*, to develop pharmacological drugs against radiation-induced ototoxicity. However, pharmacological agents have rarely been tested in clinical trials. ROS scavenging agents were found to have a radioprotective role in the auditory milieu. For instance, L-N-Acetylcysteine was shown to protect OC-k38 auditory hair cell line by promoting survival and inhibiting apoptosis 72 hours after irradiation [50]. In addition, aminothiol prc-210 alleviated inflammation and spinal ganglion injury in guinea pigs and consequently promoted better ABR responses in comparison with the control group [51]. Similarly, the synthetic steroid dexamethasone protected organ of Corti rat explants from irradiation as it reduced the levels of oxidative stress and apoptosis in a dose-dependent manner [52].

Other agents have been investigated for radioprotection in the auditory system outside the oxidative stress context. Epicatechin, a green tea extract component, abrogated apoptosis and preserved mitochondrial membrane potential after radiation exposure in HEI-OC1 and UB-OC1 cell lines by targeting the JUN and ERK pathways [53]. Piracetam, a derivative of gamma-aminobutyric acid, was found to abrogate the degeneration of the spinal ganglion, stria vascularis and outer auditory hair cell in a fractionated irradiation guinea pig model [54]. The role of metformin was also investigated in radiation-induced hearing impairment; however, no otoprotective role was elucidated for the drug [55]. Alternatively, miRNA-207 stood out as a microRNA key player in radiation-induced damage in HEI-OC1 cell line following microarray analysis [56].

Sensorineural hearing loss is further exacerbated in patients receiving chemoradiotherapy, specifically with concomitant administration of cisplatin, a well-known ototoxic agent [57, 58]. Much experimental data regarding treatment-related hearing impairment have been obtained from chemoradiation or chemotherapy-induced ototoxicity, in contrast with those who used ionising radiation solely. SNHL was found to be cisplatin and radiation dose-dependent and was associated with age, tobacco and alcohol consumption [59, 60]. Combined treatment of radiation and cisplatin resulted in worse hearing thresholds compared to subjects treated to radiation alone [61]. A well-established ROS scavenger, amifostine, had disputable results regarding cisplatin mediated hearing loss and had neurotoxic side effects on its own [57, 58, 62–64]. Numerous agents have been proven effective in alleviating cisplatin-induced ototoxicity. These include agents as N-acetylcysteine, sodium thiosulphate, D-methionine, lipoic acid, ebselen, diethyldithiocarbamate, 4-methylthiobenzoic acid, sodium salicylate, neurotrophin-3, BDNF (Brain-derived neurotrophic factor), flunarizine and NOX3 RNA silencing [57, 65]. Under basal conditions, Nfatc4 double knockout mice abrogated TNF-mediated apoptosis in auditory hair cells [66]. In a sudden loss of sensorineural hearing loss, intratympanic or oral administration of steroids was effective in auditory recovery [67]. All of the previously mentioned drugs should be investigated in a radiation context in HNC under clinical trials.

As for non-pharmacological modalities, cochlear implantations were successful in recuperating hearing post-treatment [68]. This finding elicits that some auditory nerve fibres can survive radiotherapy, albeit the extensive damage that radiation exerts on the central hearing pathway and acoustic nerves. On another note, it remains unclear if hyperbaric oxygen treatment (HBOT) could be effective in retrieving hearing sense. While it has been not effective in mild auditory impairment, HBOT might contribute to hearing improvement as a part of a multidisciplinary approach in patients with severe SNHL [69].

### Dysgeusia

Similar to SNHL, dysgeusia or taste alteration is a common side effect observed in patients with HNC undergoing radiotherapy. Taste distortion is usually a complication associated with xerostomia (dry mouth), another radiation-induced side effect in HNC. Hyposalivation was suggested to be the main reason behind the loss of gustatory abilities; however, research showed that abnormalities in taste have alternative pathophysiological aspects.

Flavours are detected via taste buds localised in the tongue, which are neuroepithelial cells innervated by the glossopharyngeal IX and facial VII nerves. Gustatory messages are then transmitted to the brain stem, specifically to the rostral solitary nucleus, and further delivered to the primary gustatory cortex. There are five basic tastes (sour, salty, sweet, bitter and savoury umami) that are sensed with the evolutionary purposes of pleasure/survival (sweet, umami and salty) and defence (sour, bitter) [70]. Alteration in taste influences food intake, nutritional profile and subsequently patients’ quality of life that could generate additional unwanted complications.

Radiation-induced dysgeusia in HNC had a predominant incidence rate of 69%–93% [71–74]. Bitter and salty tastes were the most affected after irradiation [71, 75], while the sweet taste was the least endangered [74]. Sour taste loss is more common in patients undergoing direct oral cavity radiotherapy [76]. Nevertheless, there are many discrepancies through literature regarding which flavour sensing is the most affected by radiation. The observed disparities are due to variables that differed between studies such as radiation dose, cancer type and whether subjective questionnaires or objective methods were used to assess gustatory abilities [77]. A maximum manifestation of dysgeusia was observed at a dose of 40-60 Gy to the tongue area [78]. Taste impairments start at 5 weeks post-irradiation, followed by partial recovery at 6 months. Nonetheless, partial loss of taste was still noted at 2 years post-radiotherapy [78, 79]. Dysgeusia improved between 1 and 12 months and was dependent on the dose to the oral cavity and tongue [80]. Recovery time post-treatment varied with respect to tastes through literature, but the sweet flavour was the quickest to recover [79]. Furthermore, no differences in dysgeusia prevalence were noted between hyperfractionated and conventional radiotherapy [81], while IMRT was advantageous in QoL outcome [82]. An ongoing clinical trial in Royal Marsden Hospital is correlating qualitative and quantitative data for taste loss with mean of radiation doses to the oral cavity and salivary glands using detailed dosimetric data [83].

Experimental studies were developed to investigate the mechanisms behind radiation-induced dysgeusia *in vivo*. Most studies focused on the proliferation of taste bud cells. Irradiated mice showed an increase in sucrose threshold compared to control, which could be explained by the inhibition of taste bud cells’ proliferation, particularly type II cells detecting sweet flavours [84]. A pivotal molecular study established that the downregulation of genes in the Wnt/β-catenin pathway-repressed taste buds proliferation after fractionated irradiation, mimicking treatment for HNC patients [85]. Following RT, a chain of molecular events unfolds in taste buds, starting with apoptosis (1 day), cell cycle arrest (1–3 days), entry into S and M phases (5–6 days) and a reduction of differentiated taste bud cells (7 days). The established timeline shows the sudden temporary interruption of cell proliferation post-irradiation, which is resumed around one week later [86]. Furthermore, inhibition of protein deacetylase SIRT1 in taste buds organoids promoted survival after irradiation [87]. Similarly, genetic loss of the tumour suppressor protein CHK2 was found to partially abrogate p53-mediated apoptosis of taste cells [88].

Very few drugs have been developed to mitigate radiation-induced taste alterations. Zinc sulphate was investigated in this regard, with studies reporting contradictory results. Zinc is an essential nutrient with antioxidants properties and a cofactor for many enzymes present in the taste buds. It also promotes gustin synthesis, a protein essential for the regeneration and proliferation of taste buds.

A randomised trial claimed that the administration of zinc supplements improved patients’ gustatory abilities one-month post-radiotherapy [89]. On the other hand, a study noted no difference in recovery between zinc treated and placebo groups [90]. The latter was supported by another clinical trial in which zinc sulphate failed to prevent radiation-induced dysgeusia [91]. A recent clinical trial completed in 2019 in Dow University aims to further examine the role of zinc in radiation-induced dysgeusia. However, no results have emerged from this study yet [92]. Amifostine fell short in relieving taste alterations following RT, which were surprisingly more common in the agent-treated arm compared to control in one study [93].

Medicinal herbs were also considered for treating taste alterations. Prophylactic intravenous administration of mint oil, Mint spicata, pre-radiation was found to alleviate taste aversion in rats [94]. Similar results have been uncovered with intravenous administration of a ginger extract Zingiber officinale that was found to reduce lipid peroxidation and increase superoxide anion scavenging [95].

### Dysosmia

Radiation-induced olfactory dysfunctions in patients with head and neck cancer remain poorly studied. This can be attributed to (1) the rare occurrence of nasal cavity and paranasal tumours (3% of HNC malignancies) [96], (2) the upper anatomical localisation of the olfactory milieu which is spared from radiation injury when patients are treated for the more common HNC (3) and the quick regeneration of the olfactory neuroepithelium compared to other tissues [97]. Nevertheless, dysosmia is still a side effect that impacts survivors’ day-to-day life, from food intake, well-being and inability to detect safety hazard situations [98].

A systematic review reported that odour detection and identification thresholds were impaired right after radiotherapy completion and were further exacerbated if patients had concurrent chemotherapy. Recovery of odour perception was reported as early as 6 months post-treatment. Olfactory recuperation was further prolonged up to 20 months if patients received a dose higher than 10 Gy to the olfactory epithelium. Olfactory alterations were reported as far as 5 years post-radiotherapy. Although odour detection was heavily affected, smell identification was less influenced by treatment. A study reported no effect on olfactory identification threshold up to 5 years post-radiotherapy [99]. Similar to dysgeusia, the paucity of subjective and objective assessment hinders the ability to measure smell alterations [77].

Besides its direct effects on the olfactory epithelium, radiation also impacts the nasal cavity environment. Exposure to X-rays increased nasal irrigation and obstruction along with hyposmia in patients subjected to chemoradiation, with no differences reported between conventional radiotherapy and IMRT [100]. Moreover, IMRT increased the incidence of olfactory alterations and rhinosinusitis in patients with nasopharyngeal cancer [101], which was found to be associated with the extent of the tumour and nasal irrigation [102].

The aetiology of radiation-induced dysosmia has been poorly examined. One study investigated the morphological changes in olfactory neuroepithelium following radiation in adult mice. The proliferation of the olfactory stem cells has been inhibited as early as 2 hours post-irradiation, completely abolished at 24 hours and recovered 5 weeks later. Layers of the olfactory epithelium are also affected by radiation, with the cessation of mitosis, especially in the basal lamina. Interestingly, no differences regarding apoptosis were noted in the olfactory epithelium between the irradiated and control groups [103]. A reduction in neurogenesis and size of the olfactory bulb was revealed 15 days post-irradiation. This depletion was marked by a decrease in BrdU-positive cells in the glomerular and intralaminar layers, remaining unchanged up to 60 days after irradiation. Changes in the subtypes of the interneurons connecting the olfactory epithelium to the olfactory bulb were also noted, with a marked decrease of GABA-ergic subtype [104].

No radioprotective agents against dysosmia have been adopted. However, one clinical trial at Stanford University was launched in 2017 to study the effect of olfactory training in improving smell perception in patients with nasopharyngeal and paranasal sinus cancers after radiotherapy. No results have been reported from this study to date [105]. Prior studies established that olfactory training improves patients’ sense of smell over time [106–108]. For instance, exposure to 4 different odours twice a day over 8 months in patients with smell disorders enhanced olfactory function, especially in those treated with concurrent corticosteroids [106].

There are limited treatments available to ameliorate smell alterations regardless of the underlying aetiology. Nasal occlusions, topical nasal saline and oxymetazoline HCl drops have been proposed for patients experiencing dreadful smelling (phantosmia) to reduce their odour perception. Sedatives, anti-depressants and anti-epileptic drugs have been suggested in blocking the olfactory function in patients experiencing phantosmia. However, all the abovementioned lack testing in clinical trials and may exert severe unwanted side effects. Topical cocaine drops have been advocated for anaesthetising olfactory neurons but may exhibit undesired effects [109]. In addition, short-term systematic administration of corticosteroids was proposed for sinonasal smell disorders [110], along with acupuncture [111, 112], transcranial magnetic stimulation [113], caroverine [114], pentoxyphilline [115], theophylline [116], vitamin A [117], lipoic acid [118] and minocycline [119]. The role of oral zinc administration was also examined, where it was found to have regenerative properties in post-traumatic olfactory disorders [120]. Furthermore, radical and invasive solutions as surgery have been adopted. Surgical treatments are effective in annihilating connections between the olfactory bulb and the olfactory neuroepithelium to halt phantosmia. While this method treats the symptoms, it leaves the patient with permanent smelling loss or anosmia [109]. Further studies are warranted in managing radiation-induced dysosmia taking advantage of previously established treatments for smell disorders.

### Brachial plexopathies and cranial neuropathies

Brachial plexopathies and cranial neuropathies are among the late neurological complications emanating from radiotherapy in patients with HNC. Both entities manifest as radiation insult to neuronal tissues close to or within the radiation field. Intermittent and continuous neuropathies have been reported by patients with HNC undergoing radiotherapy. Neuronal pain was mainly identified in the head and oral cavity regions, followed by the shoulder and arm areas [121].

Radiation-induced peripheral neuropathies (RIPN) are first characterised by a change in neurons’ electrophysiological state. Depending on many variables, such as radiation dose, neuronal tissues involved, physiological conditions of the patient, RIPN may resolve spontaneously or worsen over several years. Following disruption in homeostasis, inflammation-mediated fibrosis may develop, leading to a fibroatrophic state with poor vascularisation and entrapment of nerve bundles. This process is orchestrated by a dramatic increase in oxidative stress along with amplified signalling of inflammatory cytokines. Neuroinflammation is stimulated as a consequence of lysosomal digestion of necrotic neural tissues by neighbouring cells [34, 122].

Brachial plexus is defined as a network of nerves emerging from the upper cervical and thoracic part of the spinal cord. It is responsible for the muscular, cutaneous and motor-sensory innervation of the shoulder, chest and upper extremity. Thereby, injury to this plexus known as radiation-induced brachial plexopathy (RIBP) could affect patients’ motor and sensory activities, along with pain, weakness and paralysis in severe cases. Current literature investigating plexopathies and neuropathies following irradiation comes mostly from case reports.

Injury of the brachial plexus was reported in 22% of patients who had received radiotherapy for HNC either post-operatively, definitively or in combination with chemotherapy. The most prevalent symptom reported was ipsilateral pain (50%), followed by numbness or tingling feeling known as paraesthesia (40%) and motor weakness or muscle atrophy (25%) [123]. However, contradictory results were reported regarding the tolerance dose of the brachial plexus. In fact, some reported no brachial plexopathies in 72% of patients receiving a dose superior to 60 Gy [124]. Moreover, only 2% of patients reported early transient radiation-induced brachial plexopathy after the seventh and eighth months of follow-up [125]. In addition, the therapeutic dose was neither a risk factor for brachial plexopathy in sequential IMRT [126]. While a systemic meta-analysis reported a low percentage of RIPB incidence in most cohort studies, it supported the association between increasing dose and the occurrence of the injury [127]. Therefore, the radiation dose and mode of delivery may be implicated in RIBP.

Radiation-induced cranial neuropathy (RICN) has been investigated as a complication following treatment in HNC. There are many discrepancies regarding the incidence rate of this injury, with some defining it as a rare outcome [128–131], while others supporting it as a more frequent one [132–134]. The inconsistencies could be explained by differences in follow-up periods with a higher incidence reported with longer follow up. In a cross-sectional study involving 317 nasopharyngeal cancer patients, 30.9% developed cranial nerve injury, more frequently in lower nerves (25.5%) compared to the upper ones (13.9%). The cumulative incidence of cranial nerve insult reached its highest at 20 years post-radiotherapy (44.5%). Overall, radiation-induced cranial nerve palsy correlated with a total dose of radiation along with chemotherapy, upper neck fibrosis and longer duration of therapy [135, 136]. Lower cranial nerves that are most vulnerable to treatment are the glossopharyngeal IX, vagus X and the hypoglossal XII [128, 133, 135]. Clinically, such injuries manifest in difficulties in speech (dysphonia), swallowing (dysphagia) and chewing (discussed further below). Upper cranial nerves are affected to a lesser extent. As a consequence, patients displayed asymmetry of facial sensitivity, facial weakness, abnormalities in eye movement or vision due to palsies in the trigeminal V, abducens VI and facial VII nerves [128, 134, 136].

Contouring both the brachial and cranial nerve bundles is used as a first-line in the prevention of both RIBP and RICN. Delineation using computed-tomography (CT) helps in localising neural tissues and subsequent sparing from radiation [137]. A combination of both magnetic resonance imaging and CT techniques leads to better contouring, as suggested for the brachial plexus [138] and the cranial nerves [139]. Radiation oncologists usually outline the brachial plexus as organ-at-risk (OAR) following consensus guidelines and contouring atlases [137]. Many guidelines have also been developed for various cranial nerves, especially for the V and IX through XII nerves as those are the most vulnerable to radiation injury [140, 141]. Although contouring nerves is essential, some cases require reducing the total dose, dose per fraction or field size to limit neuropathic collateral damage.

While management of RIBP and RICP mainly focuses on treating the symptoms such as neuropathic pain, motor and sensory weakness, a curative strategy has yet to be established. Incidentally, numerous ongoing clinical trials are focusing on managing RIPN [142–144]. To alleviate neuroinflammation, NSAIDs are used as first-line treatment, followed by the temporary use of corticosteroids due to potential side effects [145]. The use of prednisone and methylprednisone for radiation-induced neuropathic pain in HNC is the subject of a current clinical trial in MD Anderson Cancer Center [143]. Neuropathic pain is usually symptomatically treated by opioids; however, the use of anti-epileptic drugs (gabapentin) and tricyclic anti-depressants (amitryptiline) has been suggested [34, 146]. A clinical trial in Belgium will investigate further the use of gabapentin as an alternative to opioids for neuropathic pain after radiotherapy in HNC [144]. Furthermore, a randomised clinical trial showed the effectiveness of another anti-convulsant, pregabalin, in treating radiation-induced neuropathic pain in HNC patients. The administration of the anti-epileptic drug showed improvement in QoL of patients despite the manifestation of some adverse effects [147]. Apatinib, a selective tyrosine kinase inhibitor for VEGF-2, is another agent under clinical study in China in patients with radiation-induced brain injury [142]. Contrastingly, no sufficient evidence supports the use of HBOT in easing RIBP [148]. On that note, a combinational administration of pentoxyphilline-tocopherol-clodronate did not elucidate any beneficial effect in RIBP, albeit the promising effects of these drugs in other radiation-induced side effects in HNC demonstrated by the same group [149].

More severe cases may require surgical interventions as neurolysis, nerve grafting or removal of fibrotic tissue. Neurolysis may improve nerve conduction by degenerating specific targeted groups of neurons responsible for the pain manifestation. Nerve graft and neurotisation are procedures used to treat clean-cut injuries with a proximal stump available for grafting, usually done in brachial plexopathies [146]. Moreover, the physical release of entrapped nerve bundles from excessive fibrotic tissues may improve symptoms in late stages of nerve fibrosis. Nevertheless, large clinical studies are needed to determine the effectiveness of such invasive treatments, as to date, they do not deliver satisfactory results [150].

### Optic neuropathies

Optic neuropathies are potential neurologic sequelae of RT in HNC. Radiation-induced optic neuropathy (RION) is defined as an irreversible, progressive-to-abrupt ipsilateral or bilateral visual loss, months to years following irradiation. Onset culminates at 1–2.5 years [151, 152] and ranges from 10 to 58 months post-RT [153, 154]. Usually painless, RION is an uncommon side effect with an early incidence of 9%–11% which may rise up to 20% at 5–10 years following treatment [153–155]. Skull-base malignancies along with paraorbital and paranasal tumours place the anterior visual pathways, optic nerves and chiasm at risk of injury due to their proximity to treated fields.

Pathophysiological mechanisms underlying optic neuropathies are not clear. Limited evidence suggests that the main aetiology behind RION is damage to the capillary endothelium, leading to ischemic demyelination and reactive astrocytosis [156]. In a similar fashion, radiation-induced retinopathy (RIR) is postulated to be caused by injury to the vascular endothelial cells of the retina leading to occlusive retino-vasculopathy [151]. In addition, direct neuronal injury to the retina and optic nerves has also been proposed. Moreover, radiation may cause regional alterations in retinal oxygen delivery and/or metabolism [157]. Molecularly, retinal apoptosis was found to be ATM and p53-dependent in rats [158]. Treatment with ramipril, an ACE agonist, 2 weeks post-radiation mitigated RION by restoring normal visual evoked potential *in vivo* [159]. Insufficient levels of axonal transport motor proteins as dynein-1, kinesin-1 and kinesin-2 were reported in rats following a dose of 20 Gy, while no differences were found in α-tubulin, β-tubulin and SMI31 levels [160].

To date, no treatment is available to alleviate or restore visual function once visual impairment has begun. Given the rarity of RION and RIR, few therapeutic modalities have been investigated outside case report studies. Bevacizumab, an anti-vascular endothelial growth factor, improved resolution of optic disk oedema and vision, reducing papillary haemorrhages in 64% of RION patients [161, 162]. Bevacizumab proved to be effective in treating optic neuropathies outside the RT context [163]. Conversely, HBOT and oral corticosteroids were unsuccessful in ameliorating impaired vision [156, 164, 165] in contrast to one case report [166]. Discrepancies in overall treatment time with HBOT, its initiation after RT and the severity of visual acuity could explain the inconsistency of the results. Overall, very shy data support the use of HBOT in treating RION.

A combination of bevacizumab, pentoxifylline and dexamethasone was efficacious in a case report [167]. Others described a dramatic improvement in a patient’s vision treated with pentoxifylline, vitamin E and dexamethasone [168]. Pentoxifylline alone enhanced RIR in another case report [169]. This improvement might be due to the ability of pentoxifylline to increase blood flow and flexibility of erythrocytes and leukocytes. Larger clinical studies are warranted to solidify the potential therapeutic effect of these combinational treatments. Similarly, anti-coagulants such as heparin and warfarin have been suggested to treat RION. While these agents were potent in treating other neuronal injuries related to radiation, anti-coagulants failed in ameliorating RION [170].

### Dysphagia and dysphonia

Swallowing and speech impairments are common during RT for HNC patients. While such complications are usually transient, they can persist, leading to chronic dysphagia and dysphonia that impact patients’ QoL [171]. Normal swallowing is orchestrated by multiple cranial nerves in addition to a central pattern generator located in the medulla. Deglutition allows the food bolus to pass from the oral cavity to the pharynx (Vth, VIIth and XIIth cranial nerves) while shielding the larynx (involuntary, through the IXth and Xth cranial nerves) and into the oesophagus hereafter [172]. Disordered swallowing leads to aspiration and in severe cases to pneumonia [173]. Pathophysiological mechanisms behind these impairments are mostly due to radiation-mediated fibrosis and oedema that compromise the muscular activity of the larynx and pharynx. In fact, silent aspiration is frequent in irradiated HNC patients due to an ineffective or even absent cough reflex [173, 174].

The main culprit behind the fibrotic aspect of dysphagia is TGFß. Through a well-orchestrated repertoire of inflammatory cell signalling cascades, TGFß’s activation leads to the increased deposition of collagen compromising pharyngolaryngeal structures and reducing muscular motility [171, 174–176]. Alternatively, peripheral and cranial nerves that innervate the swallowing musculature are at risk of radiation damage and could trigger the neurogenic root of dysphagia.

The incidence of dysphagia varies from 15% to 55% [171, 172, 175, 177]. This broad range could be explained by the different subjective and objective assessment methods. Dysphagia is frequently underreported by either patients or physicians [175, 178]. Furthermore, the scarcity of total dose, dose per fraction and interfraction interval, and the proximity of treated fields to the pharyngolaryngeal area, contribute in extending the incidence range [171, 175]. Moreover, increased age, smoking, prophylactic feeding tubes and surgical interventions make excellent predictors for late dysphagia [173, 175]. According to a meta-analysis, swallowing problems peak at 0–3 months after RT (acute dysphagia) and usually improve at 6–12 months after the treatment [173, 179]. Chronic dysphagia could occur 1-year post-RT if there is no improvement in swallowing activity. A dose of >41 Gy with >24% volume of the irradiated larynx was associated with an increased risk of aspiration and percutaneous endoscopic gastrostomy dependency [179]. Radiation to critical key structures such as the cricopharyngeal muscle and cervical oesophagus might have a role in acute dysphagia [180]. Late dysphagia was associated with the glottic, supraglottic larynx and pharyngeal constrictors [178, 181, 182]. IMRT succeeded in limiting radiated volumes to these areas and therefore reduced aspiration risks [181, 182].

Treatment of radiation-induced dysphagia involves various types of rehabilitation to improve the flow of the bolus and reduce aspiration risk. These include neuromuscular electrical stimulation and balloon dilatation [183], tongue, pharynx and larynx exercises [184], swallowing manoeuvres [175] acupuncture [185], postural techniques [186] and diet changes [173]. All were deemed to be effective in reducing dysphagia and improving QoL. Nevertheless, none of the previously mentioned were tested in large clinical trials, and their efficacity is questionable. In fact, a clinical trial investigating the role of acupuncture in radiation-mediated dysphagia is in progress in China [187]. An additional one in Toronto will assess the effectiveness of prophylactic swallowing exercises versus intervention exercises after the occurrence of dysphagia [188].

In contrast to dysphagia, limited studies address radiation-induced dysphonia. Non-laryngeal malignancies depending on their location can disturb voice quality, while laryngeal tumours will impede both voice and speech outcomes [189]. The expected short-term outcome could be an actual improvement in voice/speech due to shrinkage of the tumour if treatment is effective. Nevertheless, dysphonia can be the result of radiation-induced fibrosis and oedema of laryngeal structures and vocal folds. This is attributed to an increase in collagen and fibronectin deposits in the lamina propria [190, 191].

Some indicated speech deterioration after therapy [179, 192, 193]. Radiation impacted the strain, nasality, roughness and pitch aspects of patients’ voices. The effect peaks at 10 weeks post-treatment, followed by gradual improvements that never reach pre-treatment levels [193]. Acute dysphonia culminates at 10 weeks post-RT followed by a subsequent downregulation that does not reach previous normal levels. The reported incidence of dysphonia varies between 10.5% and 64% depending on the assessment method and follow-up periods [179, 192, 194]. A dose to the larynx >49.4 Gy increases dysphonia risk in HNC patients [192]. Similar to previous neuropathies, IMRT alleviated dysphonia compared to conventional treatments [192, 194].

Pharmacological treatments aiming to abrogate inflammation and fibrosis of the larynx are used as first-line in treatment of dysphonia. The use of steroids, proton pump inhibitors and TNF-α have been advocated [195]. Moreover, cryotherapy promoted regeneration and reduced inflammation and fibrosis in larynxes of rabbits [196]. Thereafter, all of the abovementioned treatments should be investigated in radiation-induced dysphonia context.

Adequate hydration of the larynx has also been proposed, considering the damaging effect of radiation on laryngeal salivary glands and mucosa leading to vocal folds dryness [195, 197]. In fact, Substance P, bombesin and enkephalin are upregulated in the innervation of these glands, suggesting a possible neurogenic root for voice impairment [198, 199]. Management of dysphonia includes non-pharmacological treatment modalities such as speech therapy. The latter aims to improve the flexibility of the larynx and to maximise phonation and articulation. Nonetheless, mixed data has been published regarding its effectiveness in treating speech impairments [195, 200–202]. In addition, multiple methods of surgical voice rehabilitation have been reported to ameliorate dysphonia in case reports [203, 204]. Recently, treating radiation-induced dysphonia with pulse dye laser has been the subject of a clinical trial in the United States [205].

## Conclusion

Radiotherapy is undoubtedly one of the major contributors to managing HNC. Nevertheless, even with the most advanced radiation techniques, damage to neighbouring healthy tissues is inevitable. In this regard, radiation-induced neuropathy in HNC showed to be a multifactorial pathological process that takes many shapes depending on the treatment modality, anatomical location and patients’ physiological conditions. Currently available medical options are modest in easing side effects, and preventive measures are successful to a certain extent. The potency and efficiency of drug-based findings should be interpreted in their ability to selectively radioprotect normal neuronal tissues while sparing tumours in HNC. Further research is urgently needed to mitigate and understand the different mechanisms underlying neural injuries following radiotherapy in HNC. In fact, current ongoing clinical trials tackling this subject are shy in numbers (table 1). ROS scavengers should be investigated as first-line radioprotective drugs. Several promising molecular venues can be further studied for mitigation of radiation neuropathies. For instance, a better knowledge of apoptotic and neuroinflammation pathways could shed light on encouraging outlooks in halting neuronal damage. Although not discussed in this review, preclinical studies could focus on apprehending neurogenesis, as it might help in improving neuroregeneration in radiation-induced neuropathies. Future research should also benefit from previously established treatments for nerve injury models unrelated to radiation. The efficacity of these interventions ought to be investigated in managing neuropathies associated with RT in HNC under clinical trials.

## Conflicts of interest

The authors declare no conflict of interest.

## Funding

The current work has no funding support.

## Figures and Tables

**Table 1.** List of recent clinical trials involved in investigating approaches in managing radiation-induced neuropathies.

Trial title	Identifier	Sponsor	Type of study	Primary treatment	Endpoint status
Gustatory Function Following Radiotherapy in Head and Neck Cancer [1]	NCT03738657	Royal Marsden NHS Foundation Trust	Cross-sectional	N/A	Recruiting
Efficacy of Zinc on Concurrent Chemo-radiotherapy Induced Taste Alterations [2]	NCT03824925	Dow University of Health Sciences	Double-blind randomised controlled trial – Phase III	*Placebo *Zinc sulfate 220mg	Completed
Olfactory Training in Improving Sense of Smell After Radiation Therapy in Patients With Paranasal Sinus or Nasopharyngeal Cancer [3]	NCT03049358	Stanford University	Single blinded randomised trial – Phase I	*Olfactory training	Terminated
Effect and Safety of Apatinib on Radiation-Induced Brain Injury [4]	NCT04152681	Sun Yat-Sen Memorial Hospital of Sun Yat-Sen University	Single group assessment – Phase II	*Apatinib 250 mg	Recruiting
Stop LCNP: High Dose Steroid Therapy for Late Radiation-Associated Lower Cranial Neuropathy [5]	NCT04151082	M.D. Anderson Cancer Center	Single group assessment – Phase I/II	*Methylprednisolone *Prednisone	Recruiting
Study of Efficacy and Safety of Gabapentin to Reduce the Need for Strong Opioid Use in Head and Neck Cancer Patients. (stREnGTH) [6]	NCT03747562	University Hospital, Ghent	Multi-center double- blinded randomised controlled trial – Phase III	*Placebo *Gabapentin 100 mg	Not yet recruiting
Efficacy of Acupuncture in Radiotherapy-induced Dysphagia in Patients With Head and Neck Cancer [187]	NCT03336775	Sun Yat-Sen Memorial Hospital of Sun Yat-Sen Universit	Single blinded randomized trial	*Acupuncture *Methylprednisolone 80 mg	Recruiting
PRO-ACTIVE: Prophylactic Swallow Intervention for Patients Receiving Radiotherapy for Head and Neck Cancer [188]	NCT03455608	University Health Network, Toronto	Randomized	*Swallowing Training	Recruiting
Pulsed Dye Laser in Treating Patients With Post Radiation Dysphonia [205]	NCT02198131	Wake Forest University Health Sciences	Single group assessment	*Pulse Dye Laser Surgery	Completed

## References

[1] Bray F, Ferlay J, Soerjomataram I (2018). Global cancer statistics 2018: GLOBOCAN estimates of incidence and mortality worldwide for 36 cancers in 185 countries. CA Cancer J Clin.

[2] Minicucci EM, Da Silva GN, Salvadori DMF (2014). Relationship between head and neck cancer therapy and some genetic endpoints. World J Clin Oncol.

[3] Borras JM, Barton M, Grau C (2015). The impact of cancer incidence and stage on optimal utilization of radiotherapy: methodology of a population based analysis by the ESTRO-HERO project. Radiother Oncol.

[4] Lumniczky K, Szatmári T, Sáfrány G (2017). Ionizing radiation-induced immune and inflammatory reactions in the brain. Front Immunol.

[5] Scaravilli F, Love S, Myers R (1986). X-irradiation impairs regeneration of peripheral nerve across a gap. J Neurocytol.

[6] Kinsella TJ, Deluca AM, Barnes M (1991). Threshold dose for peripheral neuropathy following intraoperative radiotherapy (Iort) in a large animal model. Int J Radiat Oncol Biol Phys.

[7] Fishel ML, Vasko MR, Kelley MR (2007). DNA repair in neurons: so if they don’t divide what’s to repair?. Mutat Res - Fundam Mol Mech Mutagen.

[8] Balentova S, Adamkov M (2015). Molecular, cellular and functional effects of radiation-induced brain injury: a review. Int J Mol Sci.

[9] Gobbel GT, Bellinzona M, Vogt AR (1998). Response of postmitotic neurons to x-irradiation: implications for the role of DNA damage in neuronal apoptosis. J Neurosci.

[10] Chien L, Chen WK, Liu ST (2015). Low-dose ionizing radiation induces mitochondrial fusion and increases expression of mitochondrial complexes I and III in hippocampal neurons. Oncotarget.

[11] Lu K, Zhang C, Wu W (2015). Rhubarb extract has a protective role against radiation-induced brain injury and neuronal cell apoptosis. Mol Med Rep.

[12] Shabeeb D, Musa AE, Keshavarz M (2019). Histopathological and functional evaluation of radiation-induced sciatic nerve damage: melatonin as radioprotector. Medicina.

[13] Zhang Y, Cheng Z, Wang C (2016). Neuroprotective effects of kukoamine a against radiation-induced rat brain injury through inhibition of oxidative stress and neuronal apoptosis. Neurochem Res.

[14] Tikka T, Usenius T, Tenhunen M (2001). Tetracycline derivatives and ceftriaxone, a cephalosporin antibiotic, protect neurons against apoptosis induced by ionizing radiation. J Neurochem.

[15] Saeed Y, Xie B, Xu J (2014). Indirect effects of radiation induce apoptosis and neuroinflammation in neuronal SH-SY5Y cells. Neurochem Res.

[16] Saeed Y, Xie B, Xu J (2015). Glial U87 cells protect neuronal SH-SY5Y cells from indirect effect of radiation by reducing oxidative stress and apoptosis. Acta Biochim Biophys Sin (Shanghai).

[17] Noel F, Tofilon PJ (1998). Astrocytes protect against x-ray-induced neuronal toxicity in vitro. Neuroreport.

[18] Cai L, Iskander S, Cherian MG (2004). Zinc- or cadmium-pre-induced metallothionein protects human central nervous system cells and astrocytes from radiation-induced apoptosis. Toxicol Lett.

[19] Johnson MD, Xiang H, London S (1998). Evidence for involvement of Bax and p53, but not caspases, in radiation-induced cell death of cultured postnatal hippocampal neurons. J Neurosci Res.

[20] Chong MJ, Murray MR, Gosink EC (2000). Atm and Bax cooperate in ionizing radiation-induced apoptosis in the central nervous system. Proc Natl Acad Sci USA.

[21] Hatanaka H, Enokido Y (1996). Involvement of ps3 in DNA strand break-induced apoptosis in postmitotic CNS neurons. Biochem Soc Trans.

[22] Lee Y, McKinnon PJ (2000). ATM dependent apoptosis in the nervous system. Apoptosis.

[23] Morrison RS, Kinoshita Y (2000). The role of p53 in neuronal cell death. Cell Death Differ.

[24] Chow BM, Li Y, Wong CS (2000). Radiation-induced apoptosis in the adult central nervous system is p53-dependent. Cell Death Differ.

[25] Casafont I, Palanca A, Lafarga V (2011). Effect of ionizing radiation in sensory ganglion neurons: organization and dynamics of nuclear compartments of DNA damage/repair and their relationship with transcription and cell cycle. Acta Neuropathol.

[26] Tong JX, Vogelbaum MA, Drzymala RE (1997). Radiation-induced apoptosis in dorsal root ganglion neurons. J Neurocytol.

[27] Tong J, Li J, Zhang QS (2018). Delayed cognitive deficits can be alleviated by calcium antagonist nimodipine by downregulation of apoptosis following whole brain radiotherapy. Oncol Lett.

[28] Ferrer I (1996). Cell death in the normal developing brain, and following ionizing radiation, methyl-azoxymethanol acetate, and hypoxia-ischaemia in the rat. Neuropathol Appl Neurobiol.

[29] Ferrer I, Olivé M, Blanco R (1996). Selective c-Jun overexpression is associated with ionizing radiation-induced apoptosis in the developing cerebellum of the rat. Mol Brain Res.

[30] Zhang L, Huang P, Chen H (2017). The inhibitory effect of minocycline on radiation-induced neuronal apoptosis via AMPKα1 signaling-mediated autophagy. Sci Rep.

[31] Thotala DK, Hallahan DE, Yazlovitskaya EM (2012). Glycogen synthase kinase 3β inhibitors protect hippocampal neurons from radiation-induced apoptosis by regulating MDM2-p53 pathway. Cell Death Differ.

[32] Xu M, Fan Q, Zhang J (2017). NFAT3/c4-mediated excitotoxicity in hippocampal apoptosis during radiation-induced brain injury. J Radiat Res.

[33] Lee WH, Sonntag WE, Mitschelen M (2010). Irradiation induces regionally specific alterations in pro-inflammatory environments in rat brain. Int J Radiat Biol.

[34] Delanian S, Lefaix JL, Pradat PF (2012). Radiation-induced neuropathy in cancer survivors. Radiother Oncol.

[35] Rutkowski MD, Winkelstein BA, Hickey WF (2002). Lumbar nerve root injury induces central nervous system neuroimmune activation and neuroinflammation in the rat: relationship to painful radiculopathy. Spine (Phila Pa 1976).

[36] Straub JM, New J, Hamilton CD (2016). Radiation-induced fibrosis: mechanisms and implications for therapy. Radiation-induced Fibros Mech Implic Ther.

[37] Greene-Schloesser DM, Moore E, Robbins ME (2013). Molecular pathways: radiation-induced cognitive impairment. Clin Cancer Res.

[38] Sokolova IV, Schneider CJ, Bezaire M (2015). Proton radiation alters intrinsic and synaptic properties of ca1 pyramidal neurons of the mouse hippocampus. Radiat Res.

[39] Parihar VK, Limoli CL (2013). Cranial irradiation compromises neuronal architecture in the hippocampus. Proc Natl Acad Sci USA.

[40] Wu PH, Coultrap S, Pinnix C (2012). Radiation induces acute alterations in neuronal function. PLoS One.

[41] Rosi S, Andres-Mach M, Fishman KM (2008). Cranial irradiation alters the behaviorally induced immediate-early gene Arc (activity-regulated cytoskeleton-associated protein). Cancer Res.

[42] Bellone JA, Rudobeck E, Hartman RE (2015). A single low dose of proton radiation induces long-term behavioral and electrophysiological changes in mice. Radiat Res.

[43] Kempf SJ, Moertl S, Sepe S (2014). Low-dose ionizing radiation rapidly a ff ects mitochondrial and synaptic signaling pathways in murine hippocampus and cortex. J Proteome Res.

[44] Betlazar C, Middleton RJ, Banati RB (2016). The impact of high and low dose ionising radiation on the central nervous system. Redox Biol.

[45] Leoncini E, Ricciardi W, Cadoni G (2014). Adult height and head and neck cancer: a pooled analysis within the INHANCE Consortium. Head Neck.

[46] Mujica-Mota MA, Lehnert S, Devic S (2014). Mechanisms of radiation-induced sensorineural hearing loss and radioprotection. Hear Res.

[47] Strojan P, Hutcheson KA, Eisbruch A (2017). Treatment of late sequelae after radiotherapy for head and neck cancer. Cancer Treat Rev.

[48] Bortfeld T (2006). IMRT: a review and preview. Phys Med Biol.

[49] Nutting CM, Morden JP, Beasley M (2018). Results of a multicentre randomised controlled trial of cochlear-sparing intensity-modulated radiotherapy versus conventional radiotherapy in patients with parotid cancer (COSTAR; CRUK/08/004). Eur J Cancer.

[50] Low WK, Sun L, Tan MGK (2008). L-N-Acetylcysteine protects against radiation-induced apoptosis in a cochlear cell line. Acta Otolaryngol.

[51] Giese APJ, Guarnaschelli JG, Ward JA (2015). Radioprotective effect of aminothiol PrC-210 on irradiated inner ear of Guinea pig. PLoS One.

[52] Dinh C, Chen S, Padgett K (2015). Dexamethasone protects against radiation-induced loss of auditory hair cells in vitro. Otol Neurotol.

[53] Pyun JH, Kang SU, Hwang HS (2011). Epicatechin inhibits radiation-induced auditory cell death by suppression of reactive oxygen species generation. Neuroscience.

[54] Xie LH, Tang AZ, Yin SH (2012). Protective effects of n-acetylcysteine on cochlear damage occuring in guinea pigs exposed to irradiation. Natl Med J China.

[55] Mujica-Mota MA, Salehi P, Devic S (2014). Safety and otoprotection of metformin in radiation-induced sensorineural hearing loss in the guinea pig. Otolaryngol - Head Neck Surg (United States).

[56] Jin Y, Chen D, Cabay RJ (2013). Role of microRNA-138 as a potential tumor suppressor in head and neck squamous cell carcinoma. Vol. 303 Int Rev Cell Mol Biol.

[57] Sheth S, Mukherjea D, Rybak LP (2017). Mechanisms of cisplatin-induced ototoxicity and otoprotection. Front Cell Neurosci.

[58] Hyppolito MA, De Oliveira JAA, Miranda Lessa R (2005). Otoproteção da amifostina aos efeitos otótoxicos da cisplatina: Estudo em cobaias albinas por emissões otoacústicas produtos de distorção e microscopia eletrônica de varredura. Rev Bras Otorrinolaringol.

[59] Zuur CL, Simis YJ, Lansdaal PE (2007). Ototoxicity in a randomized phase III trial of intra-arterial compared with intravenous cisplatin chemoradiation in patients with locally advanced head and neck cancer. J Clin Oncol.

[60] Shorter P, Harden F, Owen R (2017). Risk profiles for sensorineural hearing loss in patients with head and neck cancer receiving cisplatin-based chemoradiation. J Med Imaging Radiat Sci.

[61] Wong KL, Song TT, Wee J (2006). Sensorineural hearing loss after radiotherapy and chemoradiotherapy: a single, blinded, randomized study. J Clin Oncol.

[62] Gurney JG, Bass JK, Onar-Thomas A (2014). Evaluation of amifostine for protection against cisplatin-induced serious hearing loss in children treated for average-risk or high-risk medulloblastoma. Neuro Oncol.

[63] Fouladi M, Chintagumpala M, Ashley D (2008). Amifostine protects against cisplatin-induced ototoxicity in children with average-risk medulloblastoma. J Clin Oncol.

[64] Gu J, Zhu S, Li X (2014). Effect of amifostine in head and neck cancer patients treated with radiotherapy: a systematic review and meta-analysis based on randomized controlled trials. PLoS One.

[65] Rybak LP, Mukherjea D, Jajoo S (2009). Cisplatin ototoxicity and protection: clinical and experimental studies Leonard. Tohoku J Exp Med.

[66] Zhang Y, Chen D, Zhao L (2019). Nfatc4 deficiency attenuates ototoxicity by suppressing TNF-mediated hair cell apoptosis in the mouse cochlea. Front Immunol.

[67] Spear SA, Schwartz SR (2011). Intratympanic steroids for sudden sensorineural hearing loss: a systematic review. Otolaryngol - Head Neck Surg.

[68] Formanek M, Czerny C, Gstoettner W (1998). Cochlear implantation as a successful rehabilitation for radiation-induced deafness. Eur Arch Oto-Rhino-Laryngol.

[69] Eryigit B, Ziylan F, Yaz F (2018). The effectiveness of hyperbaric oxygen in patients with idiopathic sudden sensorineural hearing loss: a systematic review. Eur Arch Oto-Rhino-Laryngol.

[70] Chaudhari N, Roper SD The cell biology of taste. J Cell Biol.

[71] Mossman K, Shatzman A, Chencharick J (1982). Long-term effects of radiotherapy on taste and salivary function in man. Int J Radiat Oncol Biol Phys.

[72] Schwartz LK, Weiffenbach JM, Valdez IH (1993). Taste intensity performance in patients irradiated to the head and neck. Physiol Behav.

[73] Epstein JB, Barasch A (2010). Taste disorders in cancer patients: Pathogenesis, and approach to assessment and management. Oral Oncol.

[74] Irune E, Dwivedi RC, Nutting CM (2014). Treatment-related dysgeusia in head and neck cancer patients. Cancer Treat Rev.

[75] Barbosa da Silva JL, Doty RL, Miyazaki JVMK (2019). Gustatory disturbances occur in patients with head and neck cancer who undergo radiotherapy not directed to the oral cavity. Oral Oncol.

[76] Barbosa da Silva JL, Doty RL, Miyazaki JVMK (2019). Gustatory disturbances occur in patients with head and neck cancer who undergo radiotherapy not directed to the oral cavity. Oral Oncol.

[77] Spotten LE, Corish CA, Lorton CM (2017). Subjective and objective taste and smell changes in cancer. Ann Oncol Off J Eur Soc Med Oncol.

[78] Deshpande TS, Blanchard P, Wang L (2018). Radiation-related alterations of taste function in patients with head and neck cancer: a systematic review. Curr Treat Options Oncol.

[79] Maes A, Huygh I, Weltens C (2002). De Gustibus: Time scale of loss and recovery of tastes caused by radiotherapy. Radiother Oncol.

[80] Sapir E, Tao Y, Feng F (2016). Predictors of dysgeusia in patients with oropharyngeal cancer treated with chemotherapy and intensity modulated radiation therapy. Int J Radiat Oncol Biol Phys.

[81] Sandow PL, Hejrat-Yazdi M, Heft MW (2006). Taste loss and recovery following radiation therapy. J Dent Res.

[82] Fang FM, Chien CY, Tsai WL (2008). Quality of life and survival outcome for patients with nasopharyngeal carcinoma receiving three-dimensional conformal radiotherapy vs. intensity-modulated radiotherapy—a longitudinal study. Int J Radiat Oncol Biol Phys.

[83] NCT03049358 (2020). Gustatory Function Following Radiotherapy to the Head and Neck. NCT03049358.

[84] Jewkes BC, Barlow LA, Delay ER (2018). Effect of radiation on sucrose detection thresholds of mice. Chem Senses.

[85] Gaillard D, Shechtman LA, Millar SE (2019). Fractionated head and neck irradiation impacts taste progenitors, differentiated taste cells, and Wnt/β-catenin signaling in adult mice. Sci Rep.

[86] Nguyen HM, Reyland ME, Barlow LA (2012). Mechanisms of taste bud cell loss after head and neck irradiation. J Neurosci.

[87] Guo Q, Chen S, Rao X (2019). Inhibition of SIRT1 promotes taste bud stem cell survival and mitigates radiation-induced oral mucositis in mice. Am J Transl Res.

[88] Yuan Z, Ma J, Meng X (2018). Chk2 deficiency alleviates irradiation-induced taste dysfunction by inhibiting p53-dependent apoptosis. Oral Dis.

[89] Ripamonti C, Zecca E, Brunelli C (1998). A randomized, controlled clinical trial to evaluate the effects of zinc sulfate on cancer patients with taste alterations caused by head and neck irradiation. Cancer.

[90] Halyard MY, Jatoi A, Sloan JA (2007). Does zinc sulfate prevent therapy-induced taste alterations in head and neck cancer patients? results of phase iii double-blind, placebo-controlled trial from the North Central Cancer Treatment Group (N01C4). Int J Radiat Oncol Biol Phys.

[91] Khan AH, Safdar J, Siddiqui SU (2019). Efficacy of zinc sulfate on concurrent chemoradiotherapinduced taste alterations in oral cancer patients: A double blind randomized controlled trial. Pakistan J Med Sci.

[92] NCT03824925 (2020). Efficacy of Zinc on Concurrent Chemo-radiotherapy Induced Taste Alterations. NCT03824925.

[93] Hovan AJ, Williams PM, Stevenson-Moore P (2010). A systematic review of dysgeusia induced by cancer therapies. Support Care Cancer.

[94] Jang MH, Piao XL, Kim JM (2008). Inhibition of cholinesterase and amyloid-&bgr; aggregation by resveratrol oligomers from Vitis amurensis. Phyther Res.

[95] Sharma A, Haksar A, Chawla R (2005). Zingiber officinale Rosc. modulates gamma radiation-induced conditioned taste aversion. Pharmacol Biochem Behav.

[96] Ansa B, Goodman M, Ward K (2013). Paranasal sinus squamous cell carcinoma incidence and survival based on surveillance, epidemiology, and end results data, 1973 to 2009. Cancer.

[97] Schwob JE (2002). Neural regeneration and the peripheral olfactory system. Anat Rec.

[98] Sroussi HY, Epstein JB, Bensadoun RJ (2017). Common oral complications of head and neck cancer radiation therapy: mucositis, infections, saliva change, fibrosis, sensory dysfunctions, dental caries, periodontal disease, and osteoradionecrosis. Cancer Med.

[99] Álvarez-Camacho M, Gonella S, Campbell S (2017). A systematic review of smell alterations after radiotherapy for head and neck cancer. Cancer Treat Rev.

[100] Riva G, Raimondo L, Ravera M (2015). Late sensorial alterations in different radiotherapy techniques for nasopharyngeal cancer. Chem Senses.

[101] Wang JJ, Liang KL, Twu CW (2015). Olfactory change after intensity-modulated radiotherapy for nasopharyngeal carcinoma. Int Forum Allergy Rhinol.

[102] Su Y-x, Liu L-p, and Li L (2014). Factors influencing the incidence of sinusitis in nasopharyngeal carcinoma patients after intensity-modulated radiation therapy. Eur Arch Oto-Rhino-Laryngol.

[103] Cunha C, Hort Y, Shine J (2012). Morphological and behavioural changes occur following the X-ray irradiation of the adult mouse olfactory neuroepithelium. BMC Neurosci.

[104] Díaz D, Muñoz-Castañeda R, Ávila-Zarza C (2017). Olfactory bulb plasticity ensures proper olfaction after severe impairment in postnatal neurogenesis. Sci Rep.

[105] NCT03049358 (2017). Olfactory training in improving sense of smell after radiation therapy in patients with paranasal sinus or nasopharyngeal cancer. NCT03049358.

[106] Fleiner F, Lau L (2012). Active olfactory training for the treatment of smelling disorders. Ear, Nose Throat J.

[107] Hummel T, Reden KRJ, Hähner A (2009). Effects of olfactory Training in patients with olfactory loss. Laryngoscope.

[108] Damm M, Pikart LK, Reimann H (2014). Olfactory training is helpful in postinfectious olfactory loss: a randomized, controlled, multicenter study. Laryngoscope.

[109] Leopold D (2002). Distortion of olfactory perception: diagnosis and treatment. Chem Senses.

[110] Heilmann S, Huettenbrink KB, Hummel T (2004). Local and systemic administration of corticosteroids in the treatment of olfactory loss. Am J Rhinol.

[111] Michael W (2003). Anosmia treated with acupuncture. Acupunct Med.

[112] Wen-Ping Z, Ishida T (2006). An ageusia and dysosmia patient treated by acupuncture. Chin J Integr Med.

[113] Henkin RI, Potolicchio SJ, Levy LM (2011). Improvement in smell and taste dysfunction after repetitive transcranial magnetic stimulation. Am J Otolaryngol - Head Neck Med Surg.

[114] Quint C, Temmel AFP, Hummel T (2002). The quinoxaline derivative caroverine in the treatment of sensorineural smell disorders: a proof-of-concept study. Acta Otolaryngol.

[115] Gökalp O, Yeşilkaya NK, Beşir Y (2016). Effects of pentoxifylline on blood transfusion. Anatol J Cardiol.

[116] Henkin RI, Schultz M, Minnick-Poppe L (2012). Intranasal theophylline treatment of hyposmia and hypogeusia: a pilot study. Arch Otolaryngol - Head Neck Surg.

[117] Reden J, Lill K, Zahnert T (2012). Olfactory function in patients with postinfectious and posttraumatic smell disorders before and after treatment with vitamin A: A double-blind, placebo-controlled, randomized clinical trial. Laryngoscope.

[118] Hummel T, Heilmann S, Hüttenbriuk KB (2002). Lipoic acid in the treatment of smell dysfunction following viral infection of the upper respiratory tract. Laryngoscope.

[119] Reden J, Herting B, Lill K (2011). Treatment of postinfectious olfactory disorders with minocycline: a double-blind, placebo-controlled study. Laryngoscope.

[120] Aiba T, Sugiura M, Mori J (1998). Effect of zinc sulfate on sensorineural olfactory disorder. Acta Oto-Laryngologica.

[121] Epstein JB, Wilkie DJ, Fischer DJ (2009). Neuropathic and nociceptive pain in head and neck cancer patients receiving radiation therapy. Head Neck Oncol.

[122] Vasić L (2007). Radiation-induced peripheral neuropathies: Etiopathogenesis, risk factors, differential diagnostics, symptoms and treatment. Arch Oncol.

[123] Chen AM, Hall WH, Li J (2012). Brachial plexus-associated neuropathy after high-dose radiation therapy for head-and-neck cancer. Int J Radiat Oncol Biol Phys.

[124] Platteaux N, Dirix P, Hermans R (2010). Brachial plexopathy after chemoradiotherapy for head and neck squamous cell carcinoma. Strahlentherapie und Onkol.

[125] Metcalfe E, Etiz D (2016). Early transient radiation-induced brachial plexopathy in locally advanced head and neck cancer. Wspolczesna Onkol.

[126] Thomas TO, Refaat T, Choi M (2015). Brachial plexus dose tolerance in head and neck cancer patients treated with sequential intensity modulated radiation therapy. Radiat Oncol.

[127] Yan M, Kong W, Kerr A (2019). The radiation dose tolerance of the brachial plexus: a systematic review and meta-analysis. Clin Transl Radiat Oncol.

[128] Dong Y, Ridge JA, Ebersole B (2019). Incidence and outcomes of radiation-induced late cranial neuropathy in 10-year survivors of head and neck cancer. Oral Oncol.

[129] Hutcheson KA, Yuk M, Hubbard R (2017). Delayed lower cranial neuropathy after oropharyngeal imrt: a cohort analysis and literature review.

[130] Janssen S, Glanzmann C, Yousefi B (2015). Radiation-induced lower cranial nerve palsy in patients with head and neck carcinoma. Mol Clin Oncol.

[131] Luk YS, Shum JSF, Sze HCK (2013). Predictive factors and radiological features of radiation-induced cranial nerve palsy in patients with nasopharyngeal carcinoma following radical radiotherapy. Oral Oncol.

[132] Berger PS, Bataini JP (1977). Radiation‐induced cranial nerve palsy. Cancer.

[133] Lin YS, Jen YM, Lin JC (2002). Radiation-related cranial nerve palsy in patients with nasopharyngeal carcinoma. Cancer.

[134] Rong X, Tang Y, Chen M (2012). Radiation-induced cranial neuropathy in patients with nasopharyngeal carcinoma: a follow-up study. Strahlentherapie und Onkol.

[135] Kong L, Lu JJ, Liss AL (2011). Radiation-induced cranial nerve palsy: A cross-sectional study of nasopharyngeal cancer patients after definitive radiotherapy. Int J Radiat Oncol Biol Phys.

[136] Li JC, Mayr NA, Yuh WTC (2006). Cranial nerve involvement in nasopharyngeal carcinoma: response to radiotherapy and its clinical impact. Ann Otol Rhinol Laryngol.

[137] Yi SK, Hall WH, Mathai M (2012). Validating the RTOG-endorsed brachial plexus contouring atlas: an evaluation of reproducibility among patients treated by intensity-modulated radiotherapy for head-and-neck cancer. Int J Radiat Oncol Biol Phys.

[138] Truong MT, Nadgir RN, Hirsch AE (2010). Brachial plexus contouring with CT and MR imaging in radiation therapy planning for head and neck cancer. Radiographics.

[139] Chang JTC, Lin CY, Chen TM (2005). Nasopharyngeal carcinoma with cranial nerve palsy: the importance of MRI for radiotherapy. Int J Radiat Oncol Biol Phys.

[140] Mourad WF, Young BM, Young R (2013). Clinical validation and applications for CT-based atlas for contouring the lower cranial nerves for head and neck cancer radiation therapy. Oral Oncol.

[141] Ko HC, Gupta V, Mourad WF (2014). A contouring guide for head and neck cancers with perineural invasion. Pract Radiat Oncol.

[142] NCT04152681 (2019). Effect and Safety of Apatinib on Radiation-Induced Brain Injury. NCT04152681.

[143] NCT04151082 (2020). Stop LCNP: high dose steroid therapy for late radiation-associated lower cranial neuropathy. NCT04151082.

[144] NCT03747562 (2020). Study of efficacy and safety of gabapentin to reduce the need for strong opioid use in head and neck cancer patients. NCT03747562.

[145] Smart D (2017). Radiation toxicity in the central nervous system: mechanisms and strategies for injury Reduction. Semin Radiat Oncol.

[146] Sakellariou VI, Badilas NK, Stavropoulos NA (2014). Treatment options for brachial plexus injuries. ISRN Orthop.

[147] Jiang J, Li Y, Shen Q (2019). Effect of pregabalin on radiotherapy-related neuropathic pain in patients with head and neck cancer: a randomized controlled trial. J Clin Oncol.

[148] Pritchard J, Anand P, Broome J (2001). Double-blind randomized phase II study of hyperbaric oxygen in patients with radiation-induced brachial plexopathy. Radiother Oncol.

[149] Delanian S, Chatel C, Porcher R (2011). Complete restoration of refractory mandibular osteoradionecrosis by prolonged treatment with a pentoxifylline-tocopherol-clodronate combination (PENTOCLO): a phase II trial. Int J Radiat Oncol Biol Phys.

[150] Rubin DI, Schomberg PJ, Shepherd RFJ (2001). Arteritis and brachial plexus neuropathy as delayed complications of radiation therapy. Mayo Clin Proc.

[151] Ozkaya Akagunduz O, Guven Yilmaz S, Yalman D (2017). Evaluation of the radiation dose–volume effects of optic nerves and chiasm by psychophysical, electrophysiologic tests, and optical coherence tomography in nasopharyngeal carcinoma. Technol Cancer Res Treat.

[152] Awaguchi OSK, No TAU, Akano HIT (1999). Late retinal complications of radiation therapy for nasal and paranasal malignancies : relationship between irradiated-dose area and severity. Radiat Oncol.

[153] Demizu Y, Murakami M, Miyawaki D (2009). Analysis of vision loss caused by radiation-induced optic neuropathy after particle therapy for head-and-neck and skull-base tumors adjacent to optic nerves. Int J Radiat Oncol Biol Phys.

[154] Monroe AT, Bhandare N, Morris CG (2005). Preventing radiation retinopathy with hyperfractionation. Int J Radiat Oncol Biol Phys.

[155] Bhandare N, Monroe AT, Morris CG (2005). Does altered fractionation influence the risk of radiation-induced optic neuropathy? Int J Radiat Oncol Biol Phys.

[156] Miller NR (2004). Radiation-induced optic neuropathy: still no treatment. Clin Exp Ophthalmol.

[157] Higginson DS, Sahgal A, Lawrence MV (2013). External beam radiotherapy for head and neck cancers is associated with increased variability in retinal vascular oxygenation. PLoS One.

[158] Borges HL, Chao C, Xu Y (2004). Radiation-induced apoptosis in developing mouse retina exhibits dose-dependent requirement for ATM phosphorylation of p53. Cell Death Differ.

[159] Ryu S, Kolozsvary A, Jenrow KA (2007). Mitigation of radiation-induced optic neuropathy in rats by ACE inhibitor ramipril: importance of ramipril dose and treatment time. J Neurooncol.

[160] Yang J, Li Q, Han D (2020). Radiation-induced impairment of optic nerve axonal transport in tree shrews and rats monitored by longitudinal manganese-enhanced MRI. Neurotoxicology.

[161] Finger PT, Chin KJ (2012). Antivascular endothelial growth factor bevacizumab for radiation optic neuropathy: secondary to plaque radiotherapy. Int J Radiat Oncol Biol Phys.

[162] Finger PT (2006). Anti-VEGF Bevacizumab (Avastin®) for radiation optic neuropathy. Br Rep.

[163] Sherman JH, Aregawi DG, Lai A (2009). Optic neuropathy in patients with glioblastoma receiving bevacizumab. Neurology.

[164] Roden D, Bosley TM, Fowble B (1990). Delayed radiation injury to the retrobulbar optic nerves and chiasm: clinical syndrome and treatment with hyperbaric oxygen and corticosteroids. Ophthalmology.

[165] Malik A, Golnik K (2012). Hyperbaric oxygen therapy in the treatment of radiation optic neuropathy. J Neuro-Ophthalmol.

[166] Borruat FX, Schatz NJ, Glaser JS (1993). Visual recovery from radiation-induced optic neuropathy: the role of hyperbaric oxygen therapy. J Clin Neuroophthalmol.

[167] Farooq O, Lincoff NS, Saikali N (2012). Novel treatment for radiation optic neuropathy with intravenous bevacizumab. J Neuro-Ophthalmol.

[168] Chahal HS, Lam A, Khaderi SK (2013). Is pentoxifylline plus vitamin E an effective treatment for radiation-induced optic neuropathy?. J Neuro-Ophthalmol.

[169] Gupta P, Meisenberg B, Amin P (2001). Radiation retinopathy: the role of pentoxifylline. Retina.

[170] Weintraub JA, Bennett J, Gaspar LE (2011). Successful treatment of radiation-induced optic neuropathy. Pract Radiat Oncol.

[171] Langmore SE, Krisciunas GP (2010). Dysphagia after radiotherapy for head and neck cancer: etiology, clinical presentation, and efficacy of current treatments. Perspect Swallowing Swallowing Disord.

[172] Manikantan K, Khode S, Sayed SI (2009). Dysphagia in head and neck cancer. Cancer Treat Rev.

[173] Murphy BA, Gilbert J (2009). Dysphagia in head and neck cancer patients treated with radiation: assessment, sequelae, and rehabilitation. Semin Radiat Oncol.

[174] Nguyen NP, Moltz CC, Frank C (2004). Dysphagia following chemoradiation for locally advanced head and neck cancer. Ann Oncol.

[175] Platteaux N, Dirix P, Dejaeger E (2019). Dysphagia in head and neck cancer patients treated with chemoradiotherapy. Dysphagia.

[176] King SN, Dunlap NE, Tennant PA (2016). Pathophysiology of radiation-induced dysphagia in head and neck cancer. Dysphagia.

[177] Rinkel RN, Verdonck-de Leeuw IM, Doornaert P (2016). Prevalence of swallowing and speech problems in daily life after chemoradiation for head and neck cancer based on cut-off scores of the patient-reported outcome measures SWAL-QOL and SHI. Eur Arch Oto-Rhino-Laryngol.

[178] Mortensen HR, Jensen K, Aksglæde K (2013). Late dysphagia after IMRT for head and neck cancer and correlation with dose-volume parameters. Radiother Oncol.

[179] Heijnen BJ, Speyer R, Kertscher B (2016). Dysphagia, speech, voice, and trismus following radiotherapy and/or chemotherapy in patients with head and neck carcinoma: review of the literature. Biomed Res Int.

[180] Alterio D, Gerardi MA, Cella L (2017). Radiation-induced acute dysphagia. Prospective observational study on 42 head and neck cancer patients. Strahlentherapie und Onkol.

[181] Eisbruch A, Schwartz M, Rasch C (2004). Dysphagia and aspiration after chemoradiotherapy for head-and-neck cancer: which anatomic structures are affected and can they be spared by IMRT?. Int J Radiat Oncol Biol Phys.

[182] Feng FY, Kim HM, Lyden TH (2007). Intensity-modulated radiotherapy of head and neck cancer aiming to reduce dysphagia: early dose-effect relationships for the swallowing structures. Int J Radiat Oncol Biol Phys.

[183] Long Y, Wu XP A randomized controlled trail of combination therapy of neuromuscular electrical stimulation and balloon dilatation in the treatment of radiation-induced dysphagia in nasopharyngeal carcinoma patients. Disabil Rehabil.

[184] Tang Y, Shen Q, Wang Y (2011). A randomized prospective study of rehabilitation therapy in the treatment of radiation-induced dysphagia and trismus. Strahlentherapie und Onkol.

[185] Lu W, Posner MR, Wayne P (2010). Acupuncture for dysphagia after chemoradiation therapy in head and beck cancer: a case series report. Integr Cancer Ther.

[186] Logemann JA, Rademaker AW, Pauloski BR (1994). Effects of postural change on aspiration in head and neck surgical patients. Otolaryngol Neck Surg.

[187] NCT03336775 (2020). Efficacy of acupuncture in radiotherapy-induced dysphagia in patients with head and neck cancer. NCT03336775.

[188] NCT03455608 (2020). PRO-ACTIVE: prophylactic swallow intervention for patients receiving radiotherapy for head and neck cancer NCT03455608. NCT03455608.

[189] Jacobi I, Van Der Molen L, Huiskens H (2010). Voice and speech outcomes of chemoradiation for advanced head and neck cancer: a systematic review. Eur Arch Oto-Rhino-Laryngol.

[190] Johns MM, Kolachala V, Berg E (2012). Radiation fibrosis of the vocal fold: from man to mouse. Laryngoscope.

[191] Berg EE, Kolachala V, Branski RC (2011). Pathologic effects of external-beam irradiation on human vocal folds. Ann Otol Rhinol Laryngol.

[192] Sanguineti G, Ricchetti F, McNutt T (2014). Dosimetric predictors of dysphonia after intensity-modulated radiotherapy for oropharyngeal carcinoma. Clin Oncol.

[193] Van Der Molen L, Van Rossum MA, Jacobi I (2012). Pre- and posttreatment voice and speech outcomes in patients with advanced head and neck cancer treated with chemoradiotherapy: Expert listeners’ and patient’s perception. J Voice.

[194] Kraaijenga SAC, Oskam IM, Van Son RJJH (2016). Assessment of voice, speech, and related quality of life in advanced head and neck cancer patients 10-years+ after chemoradiotherapy. Oral Oncol.

[195] Villari CR, Courey MS (2015). Management of dysphonia after radiation therapy. Otolaryngol Clin North Am.

[196] Gong T, Zhang C, Kang J (2019). The effects of cryotherapy on vocal fold healing in a rabbit model. Laryngoscope.

[197] Lazarus CL (2009). Effects of chemoradiotherapy on voice and swallowing. Curr Opin Otolaryngol Head Neck Surg.

[198] Lidegran M, Domeij S (1995). Irradiation influences the expression of substance P and enkephalin in the rat larynx. Cell Tissue Res.

[199] Lidegran M, Forsgren S (1999). Short- and long-term effects of irradiation on laryngeal mucosa of the rat. Acta Oncol (Madr).

[200] Tuomi L, Andréll P, Finizia C (2014). Effects of voice rehabilitation after radiation therapy for laryngeal cancer: a randomized controlled study. Int J Radiat Oncol Biol Phys.

[201] Taito M, Taito S, Banno M (2019). Voice rehabilitation for laryngeal cancer after radiotherapy: a systematic review and meta-analysis. Eur Arch Oto-Rhino-Laryngol.

[202] Van Gogh CDL, Verdonck-De Leeuw IM, Boon-Kamma BA (2006). The efficacy of voice therapy in patients after treatment for early glottic carcinoma. Cancer.

[203] Sittel C, Zorowka P, Friedrich G (2002). Surgical voice rehabilitation after laser surgery for glottic carcinoma. Ann Otol Rhinol Laryngol.

[204] Shah RR, Weinstein GS, Mirza NA (2019). Voice restoration after radiation and supracricoid partial laryngectomy by injection augmentation of the arytenoid. J Voice.

[205] NCT02198131 (2020). Pulsed dye laser in treating patients with post radiation dysphonia. NCT02198131.

